# Prognosis of bronchial asthma in children with different pulmonary function phenotypes: A real-world retrospective observational study

**DOI:** 10.3389/fped.2022.1043047

**Published:** 2023-01-09

**Authors:** Lu Liu, Hui Ma, Shuhua Yuan, Jing Zhang, Jinhong Wu, Muheremu Dilimulati, Yahua Wang, Shiyu Shen, Lei Zhang, Jilei Lin, Yong Yin

**Affiliations:** Department of Respiratory Medicine, Shanghai Children’s Medical Center, School of Medicine, Shanghai Jiao Tong University, Shanghai, China

**Keywords:** pulmonary function phenotypes, small airway dysfunction, prognosis, asthma, children

## Abstract

**Objective:**

To follow up on the changes in pulmonary function phenotypes in children with asthma in the first year after diagnosis, and explore the risk factors of poor control in children with good treatment compliance.

**Methods:**

Children who were diagnosed with asthma in the Respiratory Department of Shanghai Children's Medical Center from January 1, 2019 to December 31, 2020 and were re-examined every 3 months after diagnosis for 1 year were continuously included, regardless of gender. We collected the clinical data, analyzed clinical characteristics of the different pulmonary function phenotypes at baseline and explored risk factors of poor asthma control after 1 year of standardized treatment.

**Results:**

A total of 142 children with asthma were included in this study, including 54 (38.0%) with normal pulmonary function phenotype (NPF), 75 (52.8%) with ventilation dysfunction phenotype (VD), and 13 (9.2%) with small airway dysfunction phenotype (SAD) in the baseline. Among them, there were statistically significant differences in all spirometry parameters, age, and course of disease before diagnosis (*P* < 0.05), and a negative correlation between age (*r*^2^ = −0.33, *P *< 0.001), course of disease before diagnosis (*r*^2^ = −0.23, *P* = 0.006) and FEV_1_/FVC. After 1-year follow-up, large airway function parameters and small airway function parameters were increased, fractional exhaled nitric oxide (FeNO) was decreased, the proportion of NPF was increased, the proportion of VD was decreased (*P* < 0.05), while there was no significant difference in the proportion of SAD. After 1 year of standardized treatment, 21 patients (14.8%) still had partly controlled or uncontrolled asthma. Our results showed that the more asthma attacks occurred within 1 year (OR = 6.249, 95% CI, 1.711–22.818, *P* = 0.006), the more times SAD presented at baseline and Assessment 1–4 (OR = 3.092, 95% CI, 1.222–7.825, *P* = 0.017), the higher the possibility of incomplete control of asthma.

**Conclusion:**

About 15% of the children with good treatment compliance were still not completely controlled after 1 year of treatment, which is closely associated with persistent small airway dysfunction.

## Introduction

Bronchial asthma is the most common chronic respiratory disease in childhood and characterized by chronic airway inflammation. In recent years, its prevalence has increased significantly in both Asian and Western countries. The prevalence of asthma among children aged 0–14 in China has increased from 1.97% (2000) to 3.02% (2010) (1). Alarmingly, the prevalence of asthma among children in Shanghai even increased to 14.6% in 2019 (2). Despite advances in standardized treatment for asthma in children, about 20% of children with asthma remain uncontrolled (3). This phenomenon is not only related to poor compliance and improper use of drugs (3), but also closely related to persistent airway inflammation and airway remodeling (4). Even if airway inflammation in asthma is present throughout the bronchial tree, central airways have long been recognized as the major site of airflow limitation (5). For the past few years, much evidences have shown that small airways also play a key role in the pathogenesis of asthma (6, 7).

Small airways are defined as those with an internal diameter of less than 2 mm, which extend from the eighth generation airways to the periphery of the bronchial tree (6). Physiologically, central airway resistance accounts for 85% of total airway resistance in adults (8). In the bronchial tree, there was no significant decrease in small airway diameter, but branch multiplication, exponential increase in total cross-sectional area, and a significant decrease in airway resistance (9). Therefore, small airway dysfunction (SAD) can only be identified when it became severe and extensive, and were previously called “quiet zone” (10). Children have narrower bronchi than adults, so small airway resistance is far greater than adults, even up to 50% of total airway resistance, especially in asthmatic children (11). In addition, the relationship between SAD, clinical symptoms, and control situation in children with asthma has recently become a research hotspot. Studies have shown that SAD is closely related to frequent nocturnal symptoms (12), exercise limitations (13), airway hyperreactivity (14), acute asthma attacks, and poor control (15) in children with asthma. Unlocking the “quiet zone” is urgent.

Assessment is a key component of asthma management, and its importance has been emphasized in both Global Initiative for Asthma (GINA) and the National Asthma Education and Prevention Program (NAEPP) guidelines. Clinical symptom review and pulmonary function are the most important assessment methods in asthma follow-up. Compared with clinical asthmatic manifestation assessments, pulmonary function is more objective and certain. However, most children with asthma have normal or nearly normal forced expiratory volume in 1 s (FEV_1_), which is poorly correlated with disease control (4). We should try to mine more information from pulmonary function. Pulmonary function phenotypes classified by spirometry parameters may be a feasible way. In the current study, we used our data set to achieve three aims: (i) to analyze the clinical characteristics of different pulmonary function phenotypes at baseline, (ii) to follow up on the changes in pulmonary function phenotypes in children with asthma in the first year after diagnosis, (iii) to explore the risk factors of poor control in children with good treatment compliance, especially the significance of SAD.

## Materials and methods

### Subjects

Children diagnosed with asthma in the Respiratory Department of Shanghai Children's Medical Center from January 1, 2019 to December 31, 2020 were enrolled in this study. Asthma was diagnosed following GINA criteria (16). The inclusion criteria were as follows: (i) age >4 years old, can complete spirometry examination, regardless of gender; (ii) were diagnosed with asthma for the first time, and had no history of long-term inhaled corticosteroids (ICSs) treatment; (iii) regularly reviewed in outpatient service every 3 months in the first year after diagnosis, with good compliance, without self-adjustment of medication dose or medication discontinuation; (iv) the caregiver of the child had certain Chinese reading and writing ability, could cooperate to complete the data collection. The exclusion criteria were as follows: (i) the child lost to follow-up within 1 year after diagnosis of asthma, self-adjusted medication dose or stopped medication; (ii) in addition to regular asthma medications, the child received additional treatment, such as specific immunotherapy, biological agents or traditional Chinese medicine treatment; (iii) the child had thoracic airway malformation, respiratory, cardiovascular, rheumatic system diseases and other basic diseases that may affect the results of spirometry.

### Study design

This was a real-world retrospective observational study. The demographics and clinical characteristics of all patients, and the results of their spirometry and fractional exhaled nitric oxide (FeNO) were recorded at baseline. Pulmonary function phenotypes were determined according to their spirometry parameters, and classified into normal pulmonary function phenotype (NPF), ventilation dysfunction phenotype (VD), small airway dysfunction phenotype (SAD). NPF was defined as all parameters of forced vital capacity (FVC) pred, FEV_1_ pred and FEV_1_/FVC measuring ≥80%, and any two parameters of forced expiratory flow at 50% of forced vital capacity (FEF_50%_) pred, forced expiratory flow at 75% of forced vital capacity (FEF_75%_) pred, and forced expiratory flow between 25%–75% of vital capacity (FEF_25–75%_) pred ≥65%. VD was defined as at least one parameter in FVC pred, FEV_1_ pred, and FEV_1_/FVC measuring <80%, regardless of FEF_50%_ pred, FEF_75%_ pred, FEF_25–75%_ pred. SAD was defined as all parameters of FVC pred, FEV_1_ pred and FEV_1_/FVC measuring ≥80%, and any two parameters of FEF_50%_ pred, FEF_75%_ pred, and FEF_25–75%_ pred <65%.

**Figure 1:**
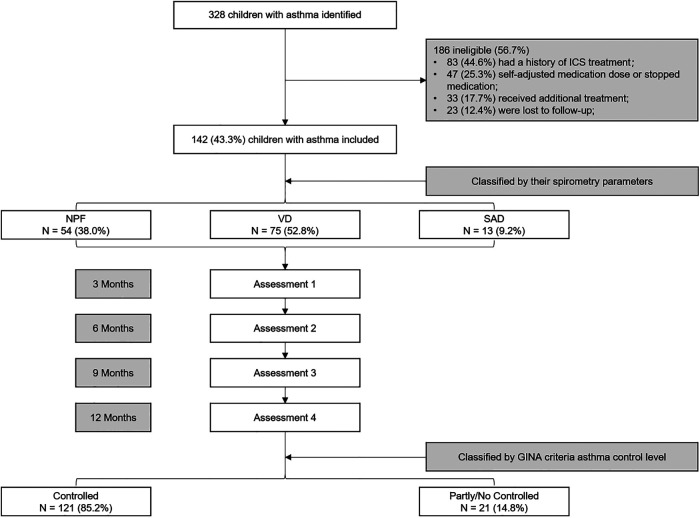
Flow diagram for the study design. NPF, normal pulmonary function phenotype; VD, ventilation dysfunction phenotype; SAD, small airway dysfunction phenotype.

All children were assessed at 3, 6, 9, and 12 months after diagnosis. Clinical symptoms, spirometry, FeNO, and asthma medication use were assessed in all of them. After 1 year of follow-up and treatment, GINA criteria were used to assess the level of asthma control (16). According to GINA criteria, asthma control level was classified into three degrees: controlled, partly controlled and uncontrolled. Children 6 to 14 years of age were in the asthma control group if they had daytime symptoms ≤2 times per week (≤1 time in children <6 years), no night awakenings due to asthma, use of reliever medications ≤2 times per week (≤1 time in children <6 years), and no limitation of activity due to asthma in the previous 4 weeks. The rest were in the partly/no controlled group. The study profile is shown in Figure 1.

### Data collection

The clinical information of children with asthma were collected from the electronic medical record system, including their birthday, sex, height, weight, date of diagnosis, history of wheezing, allergic rhinitis, atopic dermatitis, family history of allergic diseases, serum total immunoglobulin E (TIgE), and peripheral blood Eos count. The results of their spirometry and FeNO were recorded in baseline and Assessment 1–4 and these spirometry parameters were selected, including FVC% pred, FEV_1_% pred, FEV_1_/FVC, FEF_50%_ pred, FEF_75%_ pred, and FEF_25–75%_ pred. During the 1-year follow-up after diagnosis, the frequency of asthma exacerbations and respiratory infections, the use of asthma control and emergency medication were recorded in the electronic medical records by senior respiratory physicians.

### Statistical analysis

Statistical analyses were conducted using SPSS V. 26.0 (IBM SPSS Statistics, USA). Kolmogorov-Smirnov-test was used to examine the normality of the data distribution. Measurement data consistent with the normal distribution were expressed as Mean ± SD and two independent sample t-tests and ANOVA were used to compare the two groups and the three groups, respectively. The measurement data that did not conform to the normal distribution were represented by the median (interquartile range) [M (IQR)], and the Wilcoxon test and Kruskal–Wallis test were used for comparison between the two groups and the three groups, respectively. Enumeration data were expressed as the number of cases (percentage) [*n* (%)], and the chi-square test or Fisher's Exact Test was used for inter-group comparison. Spirometry parameters and FeNO were compared at baseline and after 1 year of follow-up using the two related-samples t-tests and Wilcoxon test, respectively. The changes in spirometry parameters with time were analyzed by repeated measures analysis of variance. After 1 year of follow-up, univariate and multivariate logistic regression were carried out on the risk factors that might lead to poor asthma control in children. Two-sided *P* < 0.05 indicated statistical significance.

## Results

### Demographics and baseline features

**Figure 2:**
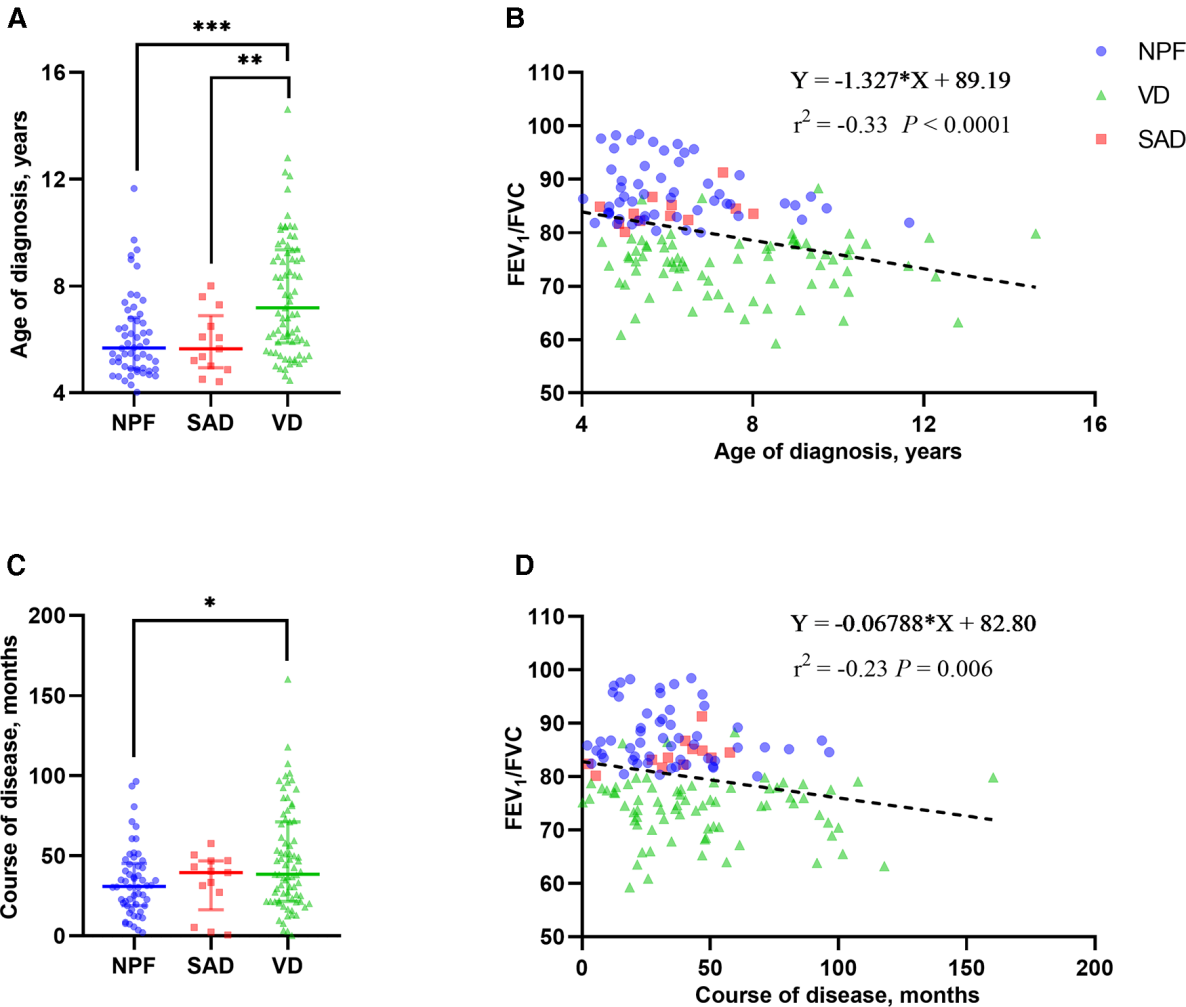
Group comparison of age of diagnosis (**A**) and course of disease (**C**) among the three phenotypes in baseline. Linear correlation analysis between FEV_1_/FVC and age of diagnosis (**B**) and course of disease (**D**). NPF, normal pulmonary function phenotype; VD, ventilation dysfunction phenotype; SAD, small airway dysfunction phenotype; FVC, forced vital capacity; FEV_1_, forced expiratory volume in 1 s. Bars indicate the median ± IQR. Group comparison among the three phenotypes: ***P < 0.001; **P < 0.01; *P < 0.05.

A total of 142 children (for 43.3% of children with asthma identified) with asthma were included in this study. Among them, 101 (71.1%) children were male, and the average age of all individuals was 6.23 (3.10) years. Based on the spirometry results at baseline, 54 children (38.0%) presented with NPF, 75 (52.8%) presented with VD, and 13 (9.2%) presented with SAD. Comparisons of demographics, clinical characterizations, and spirometry parameters between the three phenotypes are shown in Tables 1, 2. Among the three groups in baseline, there were significant differences in age (*P* < 0.001), height (*P* < 0.001), weight (*P* < 0.001), course of disease before diagnosis (*P* = 0.042) and all spirometry parameters (*P* < 0.001). In addition, the average age of VD group was older than that of NPF (adj. *P* < 0.001) and SAD (adj. *P* = 0.014) (Figure 2A), and the course of disease before diagnosis in VD group was longer than that of NPF (adj. *P* = 0.047) (Figure 2C). Tamhane's T2 multiple comparison test showed that spirometry parameters (including FVC% pred, FEV_1_% pred, FEV_1_/FVC, FEF_50%_ pred, FEF_75%_ pred and FEF_25–75%_ pred) in the NPF group were higher than those in VD and SAD, and the differences were statistically significant (*P* < 0.05). Moreover, there was a negative correlation between age (*r*^2^ = −0.33, *P* < 0.001), course of disease before diagnosis (*r*^2^ = −0.23, *P *= 0.006) and FEV_1_/FVC in all individuals (Figures 2B,D). However, no significant differences were found among the three groups in gender, allergy rhinitis, atopic dermatitis, family history of allergy, age of first wheezing episode, number of wheezes before diagnosis, serum TIgE, blood Eos count, and FeNO (*P* > 0.05).

**Table 1:** Demographics and baseline clinical characterization.

	Total	Children with NPF	Children with VD	Children with SAD
*N* (%)	142 (100)	54 (38.0)	75 (52.8)	13 (9.2)
Male, *n* (%)	101 (71.1)	34 (63.0)	57 76.0)	10 (76.9)
Female, *n* (%)	41 (28.9)	20 (37.0)	18 (24.0)	3 (23.1)
Age, years***	6.23 (3.10)	5.68 (1.92)	7.19 (3.49)	5.64 (1.95)
Height (cm)***	118.8 (18.9)	113.3 (13.2)	125.0 (24.2)	118.5 (15.3)
Wight (kg)***	22.5 (11.6)	20.4 (6.9)	26.2 (12.8)	21.4 (6.2)
BMI (kg/m^2^)	16.1 (2.7)	16.0 (2.3)	17.0 (2.9)	15.8 (3.56)
Allergy rhinitis, *n* (%)	141 (99.3)	54 (100)	74 (98.7)	13 (100)
Atopic dermatitis, *n* (%)	99 (69.7)	39 (72.2)	52 (69.3)	8 (61.5)
Family history of allergy, *n* (%)	119 (83.8)	44 (81.5)	65 (86.7)	10 (76.9)
Age at the first wheezing episode, years	3.0 (2.5)	3.0 (2.0)	3.1 (2.6)	2.8 (3.0)
Number of wheezes before diagnosis	7 (8)	6 (5)	8 (10)	6 (8)
Course of disease before diagnosis, months*	35.4 (30.0)	20.9 (26.5)	38.6 (49.6)	39.6 (30.6)
Serum TIgE, IU/ml	407 (484)	381 (530)	409 (525)	438 (297)
Blood Eos, *10^9^	0.54 (0.39)	0.50 (0.41)	0.62 (0.39)	0.51 (0.65)
FeNO, ppb	20.5 (21.0)	23.6 (16.0)	24.0 (22.0)	26.0 (24.0)

NPF, normal pulmonary function phenotype; VD, ventilation dysfunction phenotype; SAD, small airway dysfunction phenotype; BMI, body mass index; TIgE, total immunoglobulin E; FeNO, fractional exhaled nitric oxide. Data were presented as *n* (%) or median (IQR).

Comparison of the three phenotypes: ***P < 0.001; *P < 0.05.

**Table 2:** Baseline spirometry parameters among the three pulmonary function phenotypes.

	Total	Children with NPF	Children with VD	Children with SAD
FVC% pred*	102.0 ± 15.0	107.8 ± 12.8	99.3 ± 15.9	93.3 ± 8.2
FEV_1_% pred*	96.0 ± 17.6	109.8 ± 11.8	86.7 ± 15.9	91.6 ± 6.4
FEV_1_/FVC*	80.0 ± 8.4	87.5 ± 5.4	73.9 ± 5.6	83.9 ± 2.8
FEF_50%_ pred*	68.4 ± 24.5	91.2 ± 18.9	52.8 ± 15.8	64.1 ± 5.7
FEF_75%_ pred*	54.5 ± 24.3	77.0 ± 20.7	39.6 ± 14.0	47.2 ± 10.4
FEF_25–75%_ pred*	65.5 ± 30.9	89.2 ± 17.6	49.4 ± 15.2	59.8 ± 5.2

NPF, normal pulmonary function phenotype; VD, ventilation dysfunction phenotype; SAD, small airway dysfunction phenotype; FVC, forced vital capacity; FEV_1_, forced expiratory volume in 1 second; FEF_50%_, forced expiratory flow at 50% of forced vital capacity; FEF_75%_, forced expiratory flow at 75% of forced vital capacity; FEF_25–75%_, forced expiratory flow between 25 and 75% of vital capacity. Data were presented as mean ± SD. Comparison of the three phenotypes: *P < 0.001.

### Follow-up of spirometry parameters after antiasthmatic treatment

**Figure 3:**
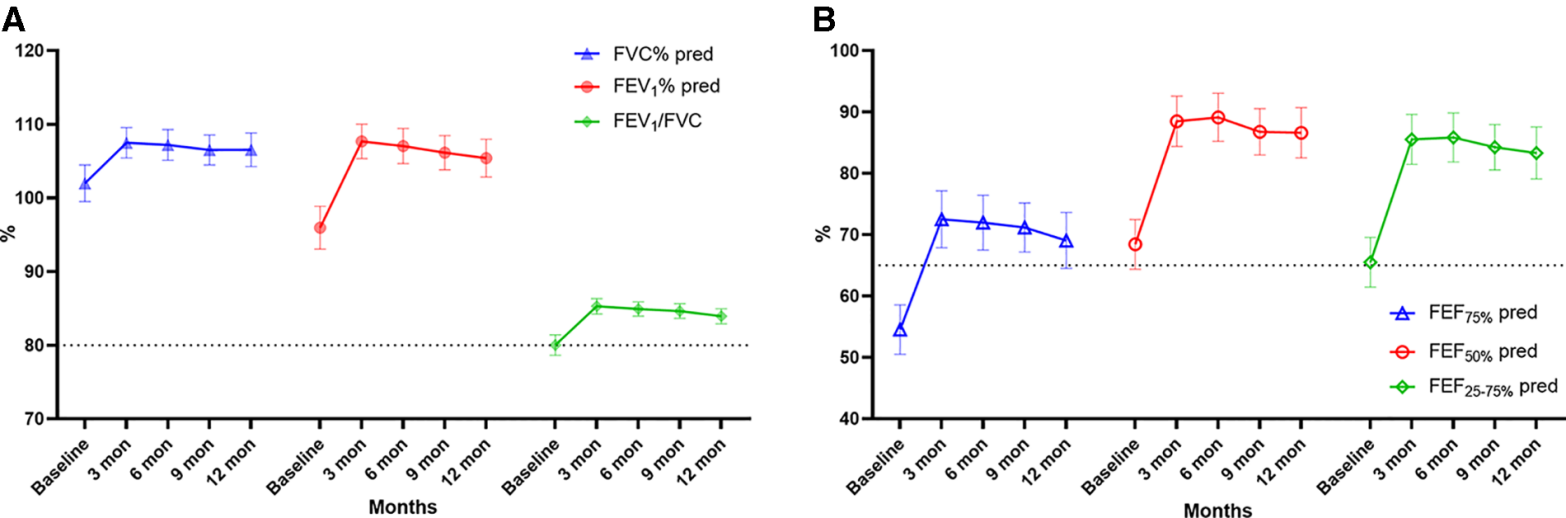
Large (**A**: FVC%, FEV_1_, FEV_1_/FVC) and small (**B**: FEF_75%_, FEF_50%_, FEF_25–75%_) airway function parameters over time. FVC, forced vital capacity; FEV_1_, forced expiratory volume in 1 s, FEF_75%_, forced expiratory flow at 75% of forced vital capacity; FEF_50%_, forced expiratory flow at 50% of forced vital capacity; FEF_25–75%_, forced expiratory flow between 25% and 75% of vital capacity. Bars indicate the mean ± 95% CI. The dashed line represents the normal values (**A**: 80%, **B**: 65%).

**Figure 4:**
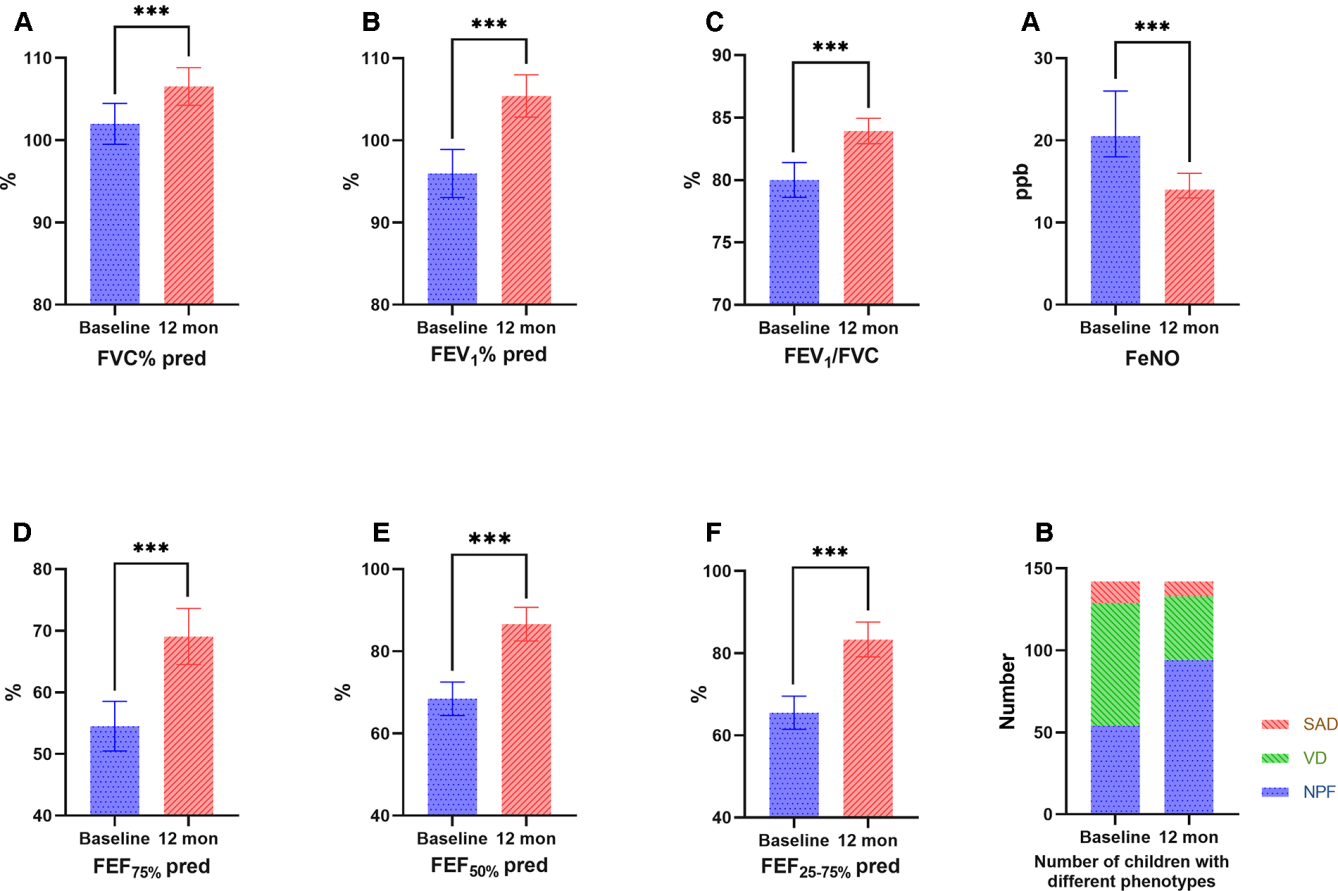
Comparison of large (**A**: FVC%, **B**: FEV_1_, **C**: FEV_1_/FVC) and small (**D**: FEF_75%_, E: FEF_50%_, F: FEF_25–75%_) airway function parameters, FeNO (**A**) and the number of children with different phenotypes (**B**) before and after treatment. FVC, forced vital capacity; FEV_1_, forced expiratory volume in 1 second, FEF_75%_, forced expiratory flow at 75% of forced vital capacity; FEF_50%_, forced expiratory flow at 50% of forced vital capacity; FEF_25–75%_, forced expiratory flow between 25% and 75% of vital capacity; FeNO, fractional exhaled nitric oxide. Bars indicate the mean ± 95% CI.

During the 1-year standardized treatment of asthma, the changes in large and small airway function parameters with time are shown in Table 3 and Figure 3. Analysis of variance of repeated measures data showed that there were statistically significant differences in large airway function parameters (FVC%, FEV_1_%, FEV_1_/FVC) (Figure 3A) and small airway function parameters (FEF_50%_, FEF_75%_, FEF_25–75%_) (Figure 3B) between baseline and Assessments 1–4 (*P* < 0.05), while there were no differences in the above indicators between Assessments 1–4. After 1 year of treatment, for all children, FVC% (mean value from 101.98% to 106.53%, *P* < 0.001) (Figure 4A), FEV_1_% (mean value from 95.96% to 105.40%, *P* < 0.001) (Figure 4B), FEV_1_/FVC (mean value from 80.01% to 83.93%, *P* < 0.001) (Figure 4C), FEF_75%_ (mean value from 54.52% to 69.05%, *P* < 0.001) (Figure 4D), FEF_50%_ (mean value from 68.44% to 86.60%, *P* < 0.001) (Figure 4E), and FEF_25–75%_ (mean value from 65.50% to 83.32%, *P* < 0.001) (Figure 4F) increased significantly, while FeNO level decreased significantly (median from 20.5 to 14.0, *P* < 0.001) (Figure 4A). Furthermore, as for the pulmonary function phenotypes, the proportion of NPF (38.0 vs. 66.2%, *P* < 0.05) increased significantly and the proportion of VD (52.8 vs. 27.5%, *P* < 0.05) decreased significantly after treatment, while the proportion of SAD (9.2% vs. 6.3%, *P* > 0.05) had no significant difference before and after treatment (Figure 4B).

**Table 3:** Spirometry parameters over time.

Months	FVC% pred	FEV_1_% pred	FEV_1_/FVC	FEF_75%_ pred	FEF_50%_ pred	FEF_25–75%_ pred
Baseline	101.98 ± 14.98	95.96 ± 17.56	80.01 ± 8.41	54.52 ± 24.28	68.44 ± 24.45	65.50 ± 24.42
3 months	107.49 ± 12.41	107.68 ± 14.05	85.28 ± 6.29	72.50 ± 28.02	88.49 ± 24.64	85.54 ± 24.49
6 months	107.21 ± 12.63	107.05 ± 14.32	84.91 ± 5.82	71.96 ± 26.85	89.13 ± 23.60	85.85 ± 24.20
9 months	106.51 ± 12.25	106.14 ± 14.10	84.64 ± 5.91	71.16 ± 24.12	86.78 ± 22.77	84.25 ± 22.37
12 months	106.53 ± 13.73	105.40 ± 15.51	83.93 ± 6.12	69.05 ± 27.46	86.60 ± 24.70	83.32 ± 25.63

FVC, forced vital capacity; FEV1, forced expiratory volume in 1 second, FEF75%, forced expiratory flow at 75% of forced vital capacity; FEF50%, forced expiratory flow at 50% of forced vital capacity; FEF25–75%, forced expiratory flow between 25% and 75% of vital capacity. Data were presented as mean ± SD.

### Prognosis after 1 year of antiasthmatic treatment

All children received standardized asthma treatment for 1 year without self-adjusting medication dose or medication discontinuation. One year later, 121 (85.2%) children achieved asthma control, while 21 (14.8%) remained partly or no controlled. The relevant risk factors for different control conditions were analyzed from the clinical and pulmonary physiological characteristics (Table 4). Compared to children with controlled asthma, the partly/no controlled individuals had lower baseline BMI percentiles (which was analyzed due to the large age range) (*P* = 0.032), more episodes of wheezes before diagnosis (*P* = 0.019), more times of asthma exacerbations during treatment (*P* < 0.001), and longer duration of emergency medication use (*P* < 0.001). However, there were no significant differences in the history of allergic diseases, family history of allergy, serum TIgE, blood Eos count, FeNO, selection and dosage of therapeutic medications between the two groups (*P* > 0.05).

**Table 4:** Risk factors for different control conditions in children with asthma: from clinical and pulmonary physiologic characteristics.

	Controlled *N* = 121 (85.2%)	Partly/No Controlled *N* = 21 (14.8%)	*t*/*Z*/*x*^2^	*P*-value
Male, *n* (%)	84 (69.4)	17 (81.0)	1.159	0.282
Female, *n* (%)	37 (30.6)	4 (19.0)		
Age, years	6.23 (3.08)	6.94 (3.29)	0.844	0.625
Baseline height (cm)	119.0 (20.5)	118.5 (16.8)	0.112	0.911
Baseline weight (kg)	22.5 (12.3)	20.0 (10.8)	1.164	0.244
Baseline BMI (kg/m^2^)	16.3 (2.8)	15.5 (2.3)	1.940	0.052
Baseline BMI percentile (%)*	62.0 (55.0)	35.0 (56.0)	2.144	0.032
Final height (cm)	125.5 (20.0)	125.5 (20.1)	0.879	0.383
Final weight (kg)	26.1 (14.3)	24.0 (14.5)	0.083	0.935
Final BMI (kg/m^2^)	16.8 (4.4)	16.2 (3.2)	1.244	0.216
Final BMI percentile (%)	70 (59.5)	67 (60.5)	0.290	0.774
Allergy rhinitis, *n* (%)	120 (99.2)	21 (100.0)	0.175	>0.999
Atopic dermatitis, *n* (%)	84 (69.4)	15 (71.4)	0.034	0.853
Family history of allergy, *n* (%)	102 (84.3)	17 (81.0)	0.148	0.749
Age at the first wheezing episode, years	3.2 (2.6)	2.7 (1.8)	0.859	0.390
Number of wheezes before diagnosis*	6 (8)	10 (9)	2.351	0.019
Course of disease before diagnosis, months	34.8 (31.0)	38.6 (39.1)	1.419	0.156
Serum TIgE, IU/ml	405 (444)	416 (661)	1.037	0.300
Blood Eos, *10^9^	5.9 (5.4)	5.7 (6.3)	0.739	0.460
Baseline FeNO, ppb	20.0 (10.0)	25.0 (24.5)	0.368	0.713
Final FeNO, ppb	13 (14.5)	18 (34.5)	1.845	0.052
Number of asthma exacerbations***				
0, *n* (%)	87 (71.9)	0 (0.0)	43.793	<0.001
1, *n* (%)	25 (20.7)	11 (52.4)		
≥2, *n* (%)	9 (7.4)	10 (47.6)		
Emergency medication use, days***	0 (2.5)	8 (15)	7.052	<0.001
Number of respiratory infections	1 (1)	1 (2)	0.226	0.821
Use of maintenance ICS				
FP pMDI, *n* (%)	15 (12.4)	2 (9.5)	0.634	0.826
FP DPI, *n* (%)	59 (48.8)	12 (57.1)		
BUD DPI, *n* (%)	47 (38.8)	7 (33.3)		
Dose of maintenance ICS (BDP; µg/d)	300 (150)	300 (150)	0.380	0.704
Combination of medication				
LARA, *n* (%)	106 (87.6)	19 (90.5)	0.140	>0.999
LTRA, *n* (%)	27 (22.3)	6 (28.6)	0.393	0.578
Intranasal CS, *n* (%)	93 (76.9)	18 (85.7)	0.822	0.414
Baseline spirometry parameters				
FVC% pred	101.4 ± 15.0	104.9 ± 14.8	0.998	0.320
FEV_1_% pred	95.5 ± 17.7	98.8 ± 16.9	0.812	0.418
FEV_1_/FVC	79.9 ± 8.4	80.1 ± 8.6	0.039	0.969
FEF_50%_ pred	68.0 ± 24.3	71.1 ± 25.9	0.543	0.588
FEF_75%_ pred	54.3 ± 24.6	55.8 ± 22.9	0.251	0.802
FEF_25–75%_ pred	65.2 ± 24.4	67.4 ± 25.1	0.384	0.701
NPF, *n* (%)	45 (37.2)	9 (42.9)	0.672	0.714
VD, *n* (%)	64 (52.9)	11 (52.4)		
SAD, *n* (%)	12 (9.9)	1 (4.8)		
Assessment 1 spirometry parameters				
FVC% pred	106.9 ± 12.2	111.1 ± 13.5	1.470	0.144
FEV_1_% pred	107.3 ± 13.8	109.7 ± 15.8	0.725	0.470
FEV_1_/FVC	85.5 ± 6.32	83.8 ± 6.1	1.164	0.246
FEF_50%_ pred	89.3 ± 25.4	83.5 ± 19.7	0.997	0.320
FEF_75%_ pred	73.8 ± 27.8	65.3 ± 28.6	1.287	0.200
FEF_25–75%_ pred	86.6 ± 24.6	79.7 ± 23.4	1.191	0.236
NPF, *n* (%)	91 (75.2)	13 (61.9)	4.389	0.086
VD, *n* (%)	26 (21.5)	5 (23.8)		
SAD, *n* (%)*	4 (3.3)	3 (14.3)		
Assessment 2 spirometry parameters				
FVC% pred	107.7 ± 12.5	104.5 ± 13.3	1.097	0.275
FEV_1_% pred	107.8 ± 14.2	102.6 ± 14.4	1.539	0.126
FEV_1_/FVC	85.1 ± 5.7	83.6 ± 6.31	1.131	0.260
FEF_50%_ pred	90.2 ± 23.7	82.9 ± 22.4	1.305	0.194
FEF_75%_ pred	75.6 ± 27.0	62.8 ± 24.5	1.712	0.089
FEF_25–75%_ pred	87.2 ± 24.0	77.9 ± 24.5	1.639	0.103
NPF, *n* (%)	94 (77.7)	14 (66.7)	3.105	0.189
VD, *n* (%)	24 (19.8)	5 (23.8)		
SAD, *n* (%)	3 (2.5)	2 (9.5)		
Assessment 3 spirometry parameters				
FVC% pred	106.3 ± 12.5	107.7 ± 11.2	0.476	0.635
FEV_1_% pred	106.1 ± 14.3	106.6 ± 13.1	0.145	0.885
FEV_1_/FVC	84.8 ± 6.1	83.9 ± 4.5	0.633	0.528
FEF_50%_ pred	87.3 ± 23.2	83.5 ± 20.4	0.706	0.482
FEF_75%_ pred	71.5 ± 24.9	69.0 ± 19.4	0.446	0.656
FEF_25–75%_ pred	84.5 ± 22.9	82.7 ± 19.5	0.340	0.734
NPF, *n* (%)	88 (72.7)	14 (66.7)	2.107	0.375
VD, *n* (%)	26 (21.5)	4 (19.0)		
SAD, *n* (%)	7 (5.8)	3 (14.3)		
Assessment 4 spirometry parameters				
FVC% pred	106.5 ± 14.1	106.9 ± 12.0	0.135	0.893
FEV_1_% pred	105.6 ± 15.8	104.5 ± 13.9	0.302	0.763
FEV_1_/FVC	84.1 ± 6.2	82.7 ± 6.0	0.976	0.331
FEF_50%_ pred	87.6 ± 25.4	80.4 ± 19.3	1.233	0.220
FEF_75%_ pred	70.3 ± 28.6	61.6 ± 18.6	1.349	0.180
FEF_25–75%_ pred	84.4 ± 26.3	76.8 ± 21.1	1.266	0.208
NPF, *n* (%)	83 (68.6)	11 (52.4)	7.003	0.029
VD, *n* (%)	33 (27.3)	6 (28.6)		
SAD, *n* (%)*	5 (4.1)	4 (19.0)		
Changes in spirometry parameters after treatment				
ΔFVC% pred	5.0 ± 14.3	1.9 ± 12.2	0.934	0.352
ΔFEV_1_% pred	10.1 ± 17.0	5.6 ± 13.7	1.145	0.254
ΔFEV_1_/FVC	4.1 ± 7.7	2.6 ± 9.1	0.797	0.427
ΔFEF_50%_ pred	19.7 ± 27.2	9.4 ± 23.0	1.639	0.103
ΔFEF_75%_ pred	16.0 ± 30.4	5.9 ± 23.0	1.463	0.146
ΔFEF_25–75%_ pred	19.3 ± 28.4	9.4 ± 23.5	1.507	0.134
Number of months of VD				
0, *n* (%)	66 (54.5)	11 (52.4)	2.366	0.306
1, *n* (%)	25 (20.7)	2 (9.5)		
≥2, *n* (%)	30 (24.8)	8 (38.1)		
Number of months of SAD***				
0, *n* (%)	102 (84.3)	15 (71.4)	19.144	<0.001
1, *n* (%)	19 (15.7)	1 (4.8)		
≥2, *n* (%)	0 (0.0)	5 (23.8)		

BMI, body mass index; TIgE, total immunoglobulin E; FeNO, fractional exhaled nitric oxide; FP pMDI, fluticasone propionate pressurized metered dose inhaler; FP DPI, fluticasone propionate dry powder inhaler; BUD DPI, budesonide dry powder inhaler; ICS, inhaled corticosteroid; BDP, beclomethasone dipropionate; LARA, Long-acting *β*2 receptor agonist; LTRA, leukotriene receptor antagonist; CS, corticosteroid; FVC, forced vital capacity; FEV1, forced expiratory volume in 1 second, FEF75%, forced expiratory flow at 75% of forced vital capacity; FEF50%, forced expiratory flow at 50% of forced vital capacity; FEF25–75%, forced expiratory flow between 25% and 75% of vital capacity; NPF, normal pulmonary function phenotype; VD, ventilation dysfunction phenotype; SAD, small airway dysfunction phenotype. Data were presented as *n* (%) or median (IQR) or mean ± SD. Comparison of the two groups: ***P < 0.001; *P < 0.05.

In addition, more attention should be paid to different pulmonary function phenotypes of the above two groups. Children in the controlled group and the partly/no controlled group showed different proportions of pulmonary function phenotypes in Assessment 4, and the main difference was that the proportion of SAD (4.1% vs. 19.0%, *P* < 0.05) was significantly higher in the latter group. Counting the number of months of VD and SAD presented at baseline and Assessment 1–4, the partly/no controlled group had more times of SAD within 1 year (*P* < 0.001) than the controlled group, while VD did not differ significantly (*P* = 0.306). However, the specific values of spirometry parameters were not statistically different between the two groups at both baseline and Assessment 1–4 (*P* > 0.05).

**Figure 5:**
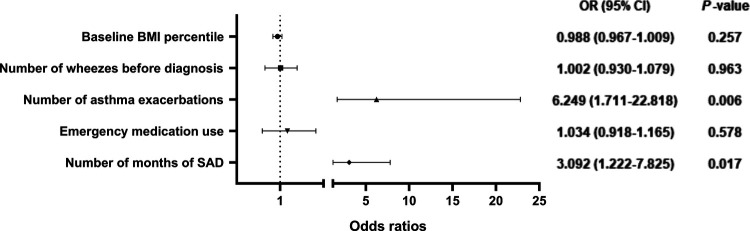
Odds ratios for risk factors for poor asthma control after 1 year of anti-asthmatic treatment. BMI, body mass index; SAD, small airway dysfunction phenotype.

The multivariate logistic regression model included the variables with statistical differences in the above univariate analysis (Figure 5). Our results showed that the more asthma attacks occurred within 1 year (OR = 6.249, 95% CI, 1.711–22.818, *P* = 0.006), the more times SAD presented at baseline and Assessment 1–4 (OR = 3.092, 95% CI, 1.222–7.825, *P* = 0.017), the higher the possibility of incomplete control of asthma after 1 year of treatment, the difference was statistically significant.

## Discussion

In this study, we found lung function impairment in children with asthma who had not yet started treatment was mainly related to their age and wheezing duration. The older the age and the longer the wheezing duration, the more severe the lung function impairment. After one year of anti-asthmatic treatment, lung function and airway inflammation were significantly improved compared to baseline, but SAD persisted, which might be closely related to poor asthma control.

There is no unified standard for the definition and diagnosis of SAD in children. In this study, the diagnosis of SAD was as follows: all parameters of FVC pred, FEV_1_ pred and FEV_1_/FVC measuring ≥80%, and any two parameters of FEF_50%_ pred, FEF_75%_ pred, and FEF_25–75%_ pred <65%. This is based on the definition of SAD in the “Expert consensus on the assessment and treatment of Small airway dysfunction in Childhood Asthma” [Respiratory Group, Pediatric Branch of Shanghai Medical Association, 2021]. Consistent with the result of current study, previous literatures using spirometry to define SAD has shown that the prevalence of SAD in asthma ranges from about 50% to 60% in adults (17) and 20% to 50% in children (6). Our study showed that the prevalence of SAD was not as high (about 10%), and this difference was related to different criteria for diagnosing SAD. For instance, SAD was defined only by FEF_25–75%_ pred <60% (18) or 65% (19) in some studies, and other studies, FEF_25–75%_ pred less than 1 times (15) or 1.64 (20) SD was used as the cut-off value to definite SAD. Many previous studies have ignored the effect of large airways on small airways. The reduction of small airway parameters is not equivalent to small airway dysfunction. When the airflow in the large airway is limited, the small airway will inevitably be affected and appear to decline. However, this condition should not be defined as SAD, but as VD.

Although spirometry is the most common way to assess SAD in children, in recent years, more methods have been applied in clinical practice, such as impulse oscillometry (IOS), imaging, etc. The IOS is a rapid, noninvasive, reproducible lung function test that measures airway resistance (R) and reactance (X) using forced oscillation techniques. The measurement of IOS is performed during normal breathing, with only passive cooperation, and is suitable for children unable to complete spirometry (21). Previous studies have shown that R_5–20_ is an effective indicator of measuring SAD (9, 22). In addition, with the development of high-resolution computed tomography (HRCT) and airway reconstruction techniques, it has become possible to evaluate small airway images. However, the resolution of HRCT is not high enough and it is difficult to directly image small airways (23). Furthermore, these methods have limited application and research in children, and their effectiveness needs to be further verified in the future.

Persistent inflammation in the small peripheral airways has recently emerged as an important contributor to poor asthma control in children with asthma (24). Our study also validated this using a 1-year follow-up of children with asthma. Our study showed that persistent SAD was a risk factor for poor asthma control. At regular follow-up visits separated by 3 months, each increase in the number of SAD was associated with a threefold increased likelihood of poor asthma control. Small airway function is closely related to asthma control in both adults (25) and children, as has been reported in the previous literatures. Rao et al. (15) performed spirometry in 744 asthmatic children aged 10 to 18 years and reviewed previous clinical information. The results showed that the number of asthma exacerbations and oral glucocorticoids were significantly increased in children with SAD. Shi et al. (26) found that school-age children with mild-to-moderate controlled asthma were at high risk for loss of control at the 8–12-week follow-up visit if there was evidence of SAD at baseline, as measured by IOS. Therefore, in clinical work, we should deepen the understanding of SAD to improve the level of asthma control in children.

Despite the availability of effective therapies, a substantial proportion of asthmatics remain poorly controlled in real life. SAD should be considered as a new target for the evaluation and treatment of asthma, especially in children with normal FEV_1_. In future studies, diagnostic criteria for SAD should be compared to determine the “gold standard”, and RCT studies should be carried out to develop drugs and inhalation devices that can treat small airway diseases. The “Quiet zone” is bound to be unlocked in the near future.

There were also some limitations to the current study. Firstly, to simply identify SAD in clinical work, this study defined SAD only by spirometry parameters (FEF_50%_, FEF_75%_, FEF_25–75%_). However, these are all functional indicators, which mismatch with the actual situation of children with asthma to a certain extent. In future studies, we should further combine IOS and/or imaging examinations to confirm SAD. In addition, the previous observational study of Pisi et al. (27) showed that FEF_25–75%_ had a high consistency with R_5–20_ in adult patients with asthma, which still needs to be verified by further studies in children. Additionally, this study was a single-center retrospective case-control study involving 142 children with asthma from Shanghai, China, who were followed up for one year. Although there are some breakthroughs compared with previous cross-sectional studies, the relationship between SAD and prognosis comes from inference. Future prospective cohort studies are needed to validate the results of our study.

## Conclusion

After 1 year of standardized anti-asthmatic treatment, SAD still existed in some children, and about 15% of them were still not completely controlled, which may be closely associated with persistent SAD. In conclusion, in childhood asthmatics with a nearly normal FEV1, persistent SAD should be considered as a potentially important pulmonary function phenotype, that can be used as a marker to judge the level of asthma control both in the clinical and research settings. Future studies should expand the sample size and extend the longitudinal follow-up time to further verify the significance of SAD in managing childhood asthma.

## Data Availability

The original contributions presented in the study are included in the article/Supplementary Material, further inquiries can be directed to the corresponding author/s.
